# A Study of Race Pacing in the Running Leg of the Japan University Triathlon Championship

**DOI:** 10.3389/fspor.2022.871727

**Published:** 2022-06-28

**Authors:** Yuto Inai, Atsushi Aoyagi, Keisuke Ishikura, Hayate Namatame, Yoshiharu Nabekura, Takao Akama

**Affiliations:** ^1^Graduate School of Sport Sciences, Waseda University, Saitama, Japan; ^2^Graduate School of Comprehensive Human Sciences, University of Tsukuba, Tsukuba, Japan; ^3^Department of Management, Faculty of Management, Josai University, Saitama, Japan; ^4^Faculty of Health and Sport Sciences, University of Tsukuba, Tsukuba, Japan; ^5^Faculty of Sport Sciences, Waseda University, Saitama, Japan

**Keywords:** performance, competition level, race analysis, pacing strategy, variation coefficient

## Abstract

Choosing an appropriate pacing strategy is important for good triathlon performance. In the Japan Student Triathlon Championship held in 2020, the men's category was divided into two groups, which was a different racing style from the previous races that all athletes start at the same time. It is highly likely that the performance level will vary as grouping was performed according to the competence of each player. The aim of this study was to understand the relationship of the total time and time of each leg between the superior performance group and the inferior performance group, as well as the difference in pacing during running in participants of the 2020 Japan University Triathlon Championship Watarase Competition, which was held under unconventional conditions. We analyzed 153 male athletes (Group A: 77; Group B: 76) who completed the race. The total race time, leg time, and average speed in each leg and its variation coefficient were evaluated based on the official results of the competition and footage recorded during the race. The results showed that the total time and leg time for each leg were significantly shorter in Group A compared to those in Group B (*p* < 0.05). In both groups, the Lap 4 run was significantly slower than those of Laps 1–3 (*p* < 0.05), while there was no significant difference in the running speed to average speed ratio across all laps between the groups (*p* < 0.05). Thus, there was a difference in running speed between the groups, but no significant difference in pacing. The results of this study serve as basic data for examining superior pacing strategies, although further studies on a wide range of competition levels are necessary.

## Introduction

A triathlon is a multidisciplinary endurance sport in which different disciplines are performed in the order of swimming, cycling, and running. Knowledge of the characteristics of the three disciplines and their relationship with the overall results is important basic data for achieving superior performance. Disciplines that predict overall performance in triathlon races have been studied at various competitive distances (Sousa et al., 2021). The standard distances used in the Summer Olympic Games are 1.5, 40, and 10 km for swimming, cycling, and running, respectively. At the 30th Olympic Games London 2012, the discipline time to total time ratio was 16.25, 53.76 and 28.93% for swimming, cycling and running, respectively (Revelles et al., 2018). Gadelha et al. (2020) reported that, in the standard distances, the results of running, which is the final leg of the three disciplines, had the greatest impact on overall performance. It has also been shown that drafting on bicycles allows for extensive energy saving on bicycles and is necessary for improved running performance (Hausswirth et al., 1999).

The energy expenditure pattern and speed distribution in a certain exercise mode are called a “pacing strategy” (Wu et al., 2014), and choosing an appropriate pacing strategy is important for good athletic performance (Abbiss and Laursen, 2008). In general, pacing strategies include negative pacing strategies, all-out pacing strategies, positive pacing strategies, even pacing strategies, parabolic-shaped pacing strategies, and variable pacing strategies (Abbiss and Laursen, 2008). Numerous studies have suggested that an even pacing strategy, in which the speed is kept at an even level, is an effective strategy for maximizing performance in endurance sports (Lambert et al., 2004; Ely et al., 2008; March et al., 2011; Hoffman, 2014; Losnegard et al., 2016). Indeed, in a 42.2-km marathon, the winning runner ran at an even pace throughout the race, while runners in the lower overall rank slowed down notably after 20–25 km (Ely et al., 2008). Furthermore, in 10- and 15-km cross-country skiing, athletes with lower final rankings (final rankings between 21st−31st and 31st−40th) were characterized by a marked decrease in speed during the race compared to athletes in the top final rankings (1st−10th) (Losnegard et al., 2016). These studies show that a strategy involving running at an even speed by reducing speed changes may be effective in endurance sports.

In studies that examined world-class elite triathletes that competed in standard-distance races where drafting was permitted, the researchers focused on sex differences and reported on pacing in all three disciplines (Vleck et al., 2008; Le Meur et al., 2009). Le Meur et al. (2009) compared pacing between men and women in the World Cup, and the results showed that women were more affected by changes in inclination than men. Moreover, Vleck et al. (2008) analyzed the cycling leg at the World Cup, and reported that the average speed and the initial speed had less impact on overall time in men than in women. Previous studies have focused on the men's and women's categories in this way and reported the impact of sex differences in pacing and performance during each disciplines on overall time.

Approximately 170 men started at the same time at the Japan University Triathlon Championship up until 2019. However, due to the spread of coronavirus disease (COVID-19), it was stipulated in 2020 that the number of participants be limited to 100 or less per race for drafting races with more than 100 participants (World Triathlon, 2020). Therefore, at the 2020 Championship, male participants were divided into two groups: Group A and Group B. The overall ranking was determined in ascending order of overall time with no distinction between Groups A and B. The 6th place winner of the same race from the previous year (venue: Kagawa Prefecture), who was seeded, elite athletes designated for strengthening and junior athletes designated for strengthening certified by the Japan Triathlon Union, and athletes in level 7 or higher in certified recorded races held by the Japan Triathlon Union were preferentially assigned to Group A. Hence, Group A is considered more likely to perform better than Group B.

Le Meur et al. (2011) examined pacing in 10-km runs at an international triathlon competition and showed that athletes with faster average run speeds tended to have less pace fluctuations throughout the run. Based on previous studies that demonstrated that keeping an even pace is conducive to superior performance in endurance sports (Lambert et al., 2004; Ely et al., 2008; March et al., 2011; Hoffman, 2014; Losnegard et al., 2016), Group A, which is expected to perform better, is more likely to show an even pace than Group B. Tomazini et al. (2015) also investigated the impact of the presence/absence of competitors on pace distribution in a 3-km run, and reported that the athletes ran at higher initial speeds in the presence of competitors than when running individually. Therefore, it is necessary to understand the proximity of athletes to each other. Although sex differences in pacing have been reported previously (Vleck et al., 2008; Le Meur et al., 2009), the between-group differences in pacing between two groups of the same sex with different start times and performance levels on the same course are unknown. Investigation of such differences will serve as basic material for formulating superior pacing strategies.

Therefore, the aim of this study was to examine the 2020 Japanese University Triathlon Championship held under unconventional conditions and understand the relationship of the total time and each leg time between the superior performance group and the inferior performance group, as well as the difference in pacing in the running leg, which has the greatest impact among the three disciplines on overall results. Our hypothesis was that the running leg has the greatest impact on overall time in both groups, that Group A performs better than Group B in all three legs, and that Group A participates in the race at speeds more even than Group B.

## Materials and Methods

### Subjects

The subjects of the analysis were 153 athletes (Group A: 77, Group B: 76) who completed the men's category of the 2020 Japan University Triathlon Championship Watarase Competition (venue: Gunma Prefecture) held on November 1, 2020. Prerequisites for participating in the race were being a recommended athlete of one of the regional blocks or being a seeded athlete. All 20 seeded athletes were assigned to Group A. Therefore, 57 athletes in Group A and 76 athletes in Group B were recommended by their respective regional blocks. Seeded athletes were those who placed in the top 6 of the same race in the previous year (Japan University Triathlon Union, 2019), those who were designated by the Japan Triathlon Union to be strengthened (Japan Triathlon Union, 2022a,b), and those who cleared the 7th level of the Japan Triathlon Union's certification record session (Japan Triathlon Union, 2017). This study was conducted with the Waseda University's academic research ethical review committee regarding procedures concerning research with human subjects (Approval Number: 2020-230) and the Ethics Committee of the University of Tsukuba (project identification code: Tai 20-122). The video recording in this study was conducted with the permission of the Japan Student Triathlon Union. The purpose and methods of this study were explained orally to the subjects, and they freely agreed to participate in this study.

### Procedure

Group A started at 10:39:58 and Group B started at 12:38:58. The weather on the day of the race was clear with a water temperature of 17°C, an air temperature of 15°C, and a wind speed of 1 m/s north (Japan University Triathlon Union, 2020). Originally, two laps (750 m per lap) were scheduled for the swimming leg. However, on the day of the race, the subjects wore wet suits and swam only one lap (750 m in total) due to the low water temperature. For the cycling leg, the subjects made six laps (6.6 km per lap) on a flat course (39.6 km in total). This race allowed drafting during the cycling leg. For the running leg, the subjects ran two laps on a flat course (9.2 km in total).

### Measuring Equipment and Data Collection

Video cameras (PanasonicHC-W950, SONY FDR-AX30) were installed on the roadside of the turning point of the running course and also at the other turning point. The speed of the subjects in sections 0–2.25 km (Lap 1), 2.25–4.611 km (Lap 2), 4.611–6.886 km (Lap 3), and 6.886–9.239 km (Lap 4) was calculated using the captured video data. The distance between each measurement point was measured using a distance measuring wheel (TRUSCOTRC-50). The official results of the competition (Japan University Triathlon Union, 2020) were used for the total time, swimming leg time, cycling leg time including transition time, split time which is the time required from the start of the swim leg to the start of the run leg, and running leg time. The variation coefficient was calculated by dividing the standard deviation of the average speed of each section by the average speed of all sections for each subject to evaluate the fluctuation in speed in each section of the running leg (Hoffman, 2014). Furthermore, we calculated the performance density for the top eight subjects in the running leg from both groups: [(running speed of the first-place subject) – (running speed of the eighth-place subject)]/(running speed of the first-place subject) ×100. Performance density expresses the difference between the speeds of the first-place subject and eighth-place subject in the running leg as a percentage of the speed of the first-place subject. The density of the top eight fastest subjects is shown (Ferro et al., 2001).

### Statistical Analysis

Each measured value is shown as the median (minimum–maximum). A Shapiro-Wilk test was performed to verify the normality of the data, and normality was rejected for most lap times. Spearman's rank correlation coefficient was used to analyze the relationship between the total time and each leg time, and the relationship between the average speed and the variation coefficient. If the coefficient is between 0 and 0.19 reflect a very weak correlation, between 0.20 and 0.39 is a weak correlation, between 0.40 and 0.59 is a moderate correlation, between 0.60 and 0.80 is considered a strong correlation and between 0.80 and 1 indicates a very strong correlation (Wealleans et al., 2021). The Mann–Whitney *U* test was used to analyze the difference between Group A and Group B. Wilcoxon signed rank test was used to analyze the difference between each lap in the same group. The effect size was evaluated as *r* (with 0.1 considered to be a small, 0.3 a medium, and 0.5 a large effect) (Fritz et al., 2012). SPSS Statistics (version 24.0) was used for statistical analysis, and the significance level was set to <5%.

## Results

Table 1 shows the Total time and each leg time. The total times for Group A and Group B were 1:48:34 (1:40:05–2:11:42) and 1:52:36 (1:45:22–2:16:33), respectively. The swimming leg of Group A and Group B were 0:10:04 (0:09:01–0:11:46) and 0:11:55 (0:09:26–0:14:08), respectively. The cycling leg for groups A and B were 1:04:02 (1:01:45–1:13:44) and 1:04:50 (1:02:22–1:13:16), respectively. The split time of Group A and Group B were 1:14:26 (1:10:52–1:24:42) and 1:15:19 (1:12:44–1:25:15), respectively. The running leg of Group A and Group B were 0:34:11 (0:29:13–0:51:52) and 0:35:51 (0:30:07–0:54:19), respectively.

**Table 1:** Total time, split time and each leg time.

	**Total time**	**Swimming leg**	**Cycling leg**	**Split time**	**Running leg**
Group A	1:48:34 (1:40:05–2:11:42)	0:10:04 (0:09:01–0:11:46)	1:04:02 (1:01:45–1:13:44)	1:14:26 (1:10:52–1:24:42)	0:34:11 (0:29:13–0:51:52)
Group B	1:52:36 (1:45:22–2:16:33)	0:11:55 (0:09:26–0:14:08)	1:04:50 (1:02:22–1:13:16)	1:15:19 (1:12:44–1:25:15)	0:35:51 (0:30:07–0:54:19)

*Data are presented as median and (minimummaximum) for each group*.

The total time and each leg time were significantly shorter in Group A compared to Group B (total time: *p* = 0.000, *r* = −0.365, swimming leg: *p* = 0.000, *r* = −0.673, cycling leg: *p* = 0.029, *r* = −0.176, split time: *p* = 0.000, *r* = −0.609, and running leg: *p* = 0.014, *r* = −0.200). The time difference between groups was 0:04:02 (total time), 0:01:51 (swimming leg), 0:00:47 (cycling leg), and 0:01:40 (running leg), with the swimming leg showing the largest difference. In addition, 45.9% of the difference in total time was accounted for by the difference in the swimming leg.

Table 2 shows the correlation coefficient between the total time and each leg time. All leg times showed a significant positive correlation with total time in both groups (*p* = 0.000). Among them, the running leg was the most strongly associated with the total time (Group A: ρ = 0.871, Group B: ρ = 0.850). The running speed in the running leg for both groups combined was 15.8 (10.2–18.9) km/h.

**Table 2:** Correlation coefficient of total time and each leg time (rho).

	**Swimming leg**	**Cycling leg**	**Split time**	**Running leg**
Group A	0.568	0.700	0.724	0.871
Group B	0.446	0.757	0.808	0.850

*p = 0.000 in all instances*.

**Figure 1:**
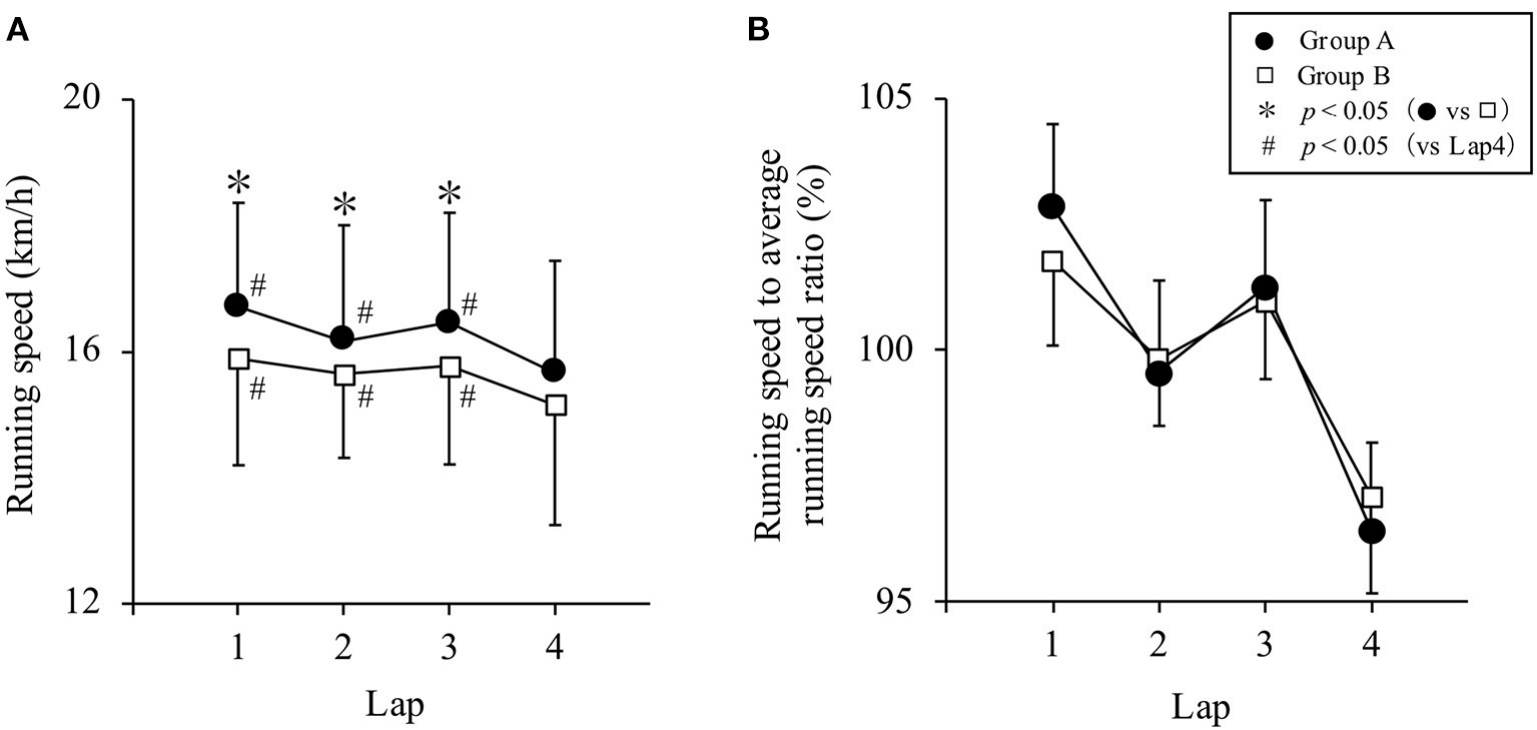
**(A)** Running speed and **(B)** running speed to average running speed ratio in the running leg. Data are presented as median and standard error for each group.

Figure 1 shows the running speed and pacing for both groups in the running leg. Group A was significantly faster in running Lap 1, Lap 2, and Lap 3 compared to group B (*p* = 0.002, 0.009, and 0.009, *r* = −0.245, −0.211, and −0.210, respectively). In both groups, running Lap 4 was significantly slower than Lap 1, Lap 2, and Lap 3 (Group A: *p* = 0.000 in all Laps, *r* = −0.692, −0.478, −0.722, Group B: *p* = 0.000, 0.001, 0.000, *r* = −0.524, −0.396, −0.649, respectively). The variation coefficient in the running leg was 3.5% (1.0–26.2) in Group A and 3.4% (0.6–4.5) in Group B, with no significant difference between the two groups (*p* = 0.570, *r* = −0.046). There was no significant difference in the running speed to average running speed ratio across all laps between groups (*p* = 0.247, 0.477, 0.585 and 0.219, *r* = −0.094, −0.058, −0.044, −0.099, respectively).

**Figure 2:**
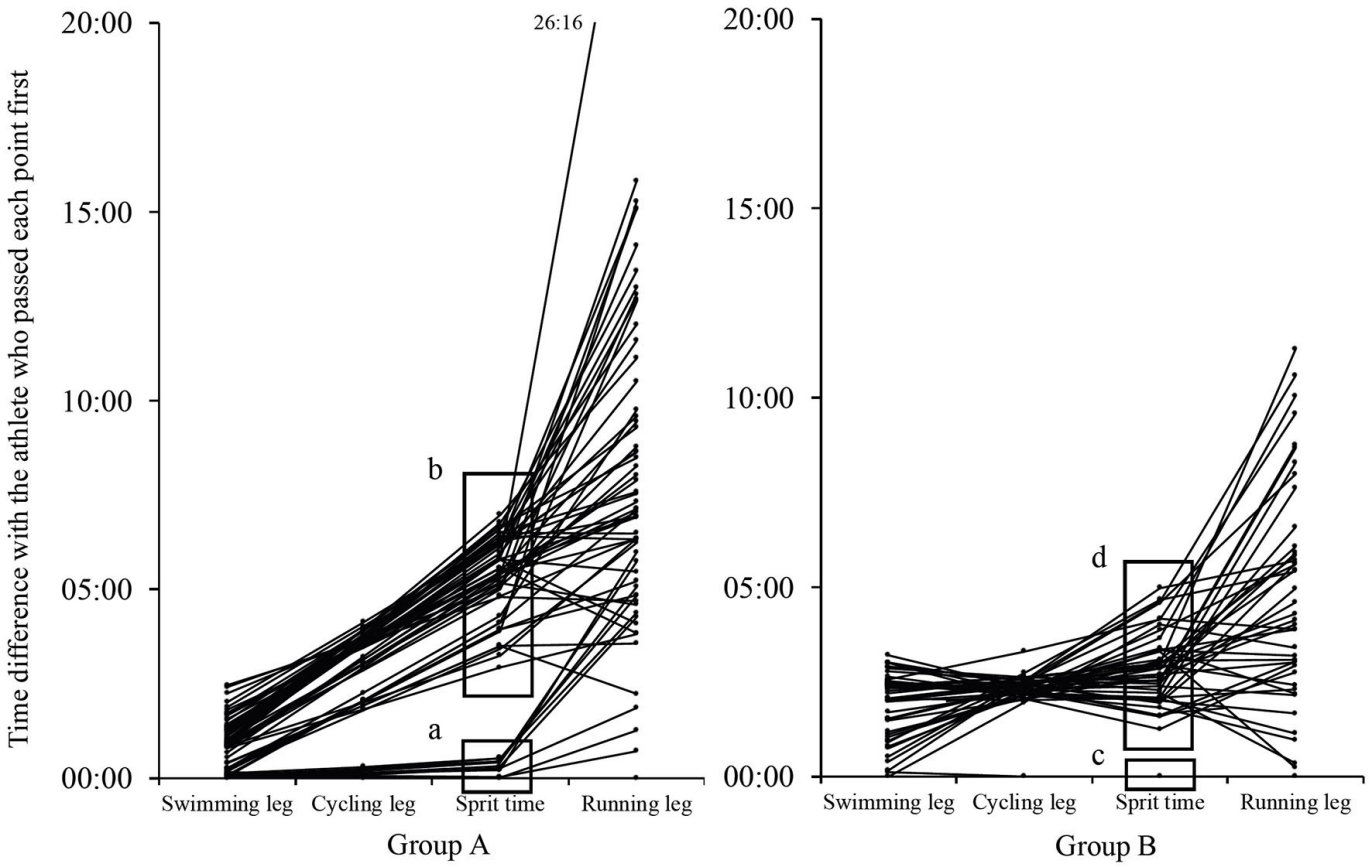
Difference in time between the athletes who passed each point first. The first pack of Group A is designated as “a” and the second pack as “b,” while the first pack of Group B is designated as “c” and the second pack as “d” at the split time. Data are presented as individual values.

Figure 2 shows the degree of crowding of athletes at the start of the running leg, focusing on athletes whose split times are within 7 min of the front of the pack. In Figure 2, the first group of Group A is designated as “a” and the second group as “b,” while the first group of Group B is designated as “c” and the second group as “d” at the split time. At the start of runs (a), (b), (c), and (d), 10 athletes started within 32 s (1 athlete every 3 s on average), 52 athletes within 4 min and 3 s (1 athlete every 5 s on average), only 1 athlete, and 43 athletes within 3 min and 44 s (1 athlete every 5 s on average), respectively. The performance density in the running leg was 4.41 and 7.23% for both groups, respectively.

## Discussion

The aim of this study was to understand the relationship between the total time and each leg time of the superior performance group and the inferior performance group, as well as the difference in pacing in the running leg in participants of the 2020 Japan University Triathlon Championship Watarase Competition, which was held under unconventional conditions. Compared to Group B, the total time and each leg time were significantly shorter in Group A, and the running time had the largest impact on the total time. These results confirmed the hypothesis that Group A would perform better than Group B. Therefore, it can be said that Group A performed better than Group B. However, there was no significant difference in the variation coefficient for the running leg between the two groups and the hypothesis that Group A would run at speeds more even than Group B was not supported. This was contrary to the report by Hoffman (2014), which suggested that keeping an even pace was a superior pacing strategy in endurance sports. The running speed in this race was 15.8 (10.2–18.9) km/h, while the running speed in the Triathlon World Cup was 18.2 (15.6–19.6) km/h (Le Meur et al., 2009). Thus, it is inferred that this race was a low-level drafting race. This study revealed that, on the same course, there was no significant difference in pacing during the running leg of a low-level drafting race between two same-sex groups with different start times and performance levels.

In this study, all leg times were positively correlated with the total time in both groups, with the running leg most strongly related to the total time. This suggests that the relationship between the disciplines was similar in both groups with respect to the total time. Our results differ from those of Sousa et al. (2021), but are in agreement with many other previous studies indicating that the total time has the largest impact on the running leg (Landers et al., 2000; Fröhlich et al., 2008; Gadelha et al., 2020). Group A was faster than Group B in total time, each lap time, and run Lap 1, Lap 2, and Lap 3. These results may be attributed to the fact that Group A included top-ranking participants of the same race from the previous year and athletes designated for strengthening, which may have resulted in higher speeds. If the competition is held under similar conditions in 2021 and onwards, athletes in Group B, which does not include top-ranking participants of the same race from the previous year or athletes designated for strengthening, may need to take care in deciding the target ranking prior to the race.

It is difficult for triathletes and coaches to evaluate whether the pace was appropriate for the athlete only by checking the official results of the competition. In this study, we examined the variation in pace with respect to the average running speed in the running leg. The results showed no significant difference in the variation coefficient or the running speed to average running speed ratio, although it was hypothesized that there would be less pace fluctuation during the running leg in Group A than in Group B. This may be because Group A is superior to Group B in terms of performance and it is presumed that the athletes were unable to keep their pace as they started the running leg with a narrow margin as seen in Group A of Figure 2. In the running leg, as shown in Figure 2, Group A started with an average interval of one person every 3 s (a) and Group B started with an average interval of one person every 5 s (d). And the performance density of the running leg was 4.41% in Group A and 7.23% in Group B. This suggests that Group A was more competitive with other athletes than Group B. As shown in Group A of Figure 2B, there was no statistical difference, but Group A may have been over-paced because Lap 1 was slightly higher and Lap 4 was slower than Group B. Therefore, the variation coefficient of Group A might not have been lower than that of Group B. In a 3-km run, the initial running speed was higher in the presence of competitors than when running alone (Tomazini et al., 2015). The athletes in Group A were at similar performance levels, and it is possible that the condition of the competitors affected pace distribution. It has been widely suggested that keeping an even pace is an effective strategy for maximizing performance in endurance sports (Lambert et al., 2004; Ely et al., 2008; March et al., 2011; Hoffman, 2014; Losnegard et al., 2016). The reason for this is that the runner's physiological demands increase and performance deteriorates as the pace becomes uneven and the energy expenditure is not kept even (Staab et al., 1992). It is therefore better to run at an even pace in this race as well. In addition, run Lap 4 was slower than Lap 1, Lap 2, and Lap 3 in both groups. Ishikura and Moriya (2017) reported that the top eight male athletes of the World Triathlon Series ran Laps 1–4 at an even pace. This race was different from elite races in that the final lap was slower than the preceding laps. It is possible that many of the athletes were overpaced as run Lap 4 was slower than Lap 1, Lap 2, and Lap 3. With respect to athletes performing at levels higher than those that participated in this race, the faster the athlete the more even their pace (Le Meur et al., 2011; Ishikura and Moriya, 2017). It is possible that there is a difference in the pacing characteristics between the world's elite athletes and the university students who were the subjects of this study. Going forward, evaluation is required to study pacing in elite races of a wide range of levels, including the Olympic Games, World Triathlon Championship Series, and Japan Championship.

This study has several limitations. First, we were only able to obtain the speed within 2.5-km sections in the running leg of this race. Tucker et al. (2006) analyzed the pacing of athletes when they achieved world records in track and field, and reported that final sprints occurred in races with a distance of 5,000 and 10,000 m based on the section speeds they calculated for every 1,000 m (Tucker et al., 2006). Final sprints are also observed before the finish in triathlon races (Le Meur et al., 2011). Therefore, the pacing in the running leg may be examined in more detail by installing video cameras at 9,000 and 9,500 m points of the running leg and analyzing the presence and degree of final sprints in future studies. We next examined pacing by analyzing the competition time but we were unable to study the reasons behind such pacing. Factors affecting pacing include temperature, oxygen concentration in the air, and muscle glycogen content (Tucker and Noakes, 2009). Therefore, evaluation of factors that affect pacing may also be required in future studies.

In conclusion, the aim of this study was to understand the relationship between the total time and each leg time, as well as the difference in pacing in the running leg by examining groups with different performance levels in a triathlon race. The results of each discipline were related to the overall results, regardless of the performance level of the group, and the results of the running leg were the most strongly associated with the overall results. For the running leg, there was a difference in performance between the groups. However, one of the findings of this study is that, despite our hypothesis that Group A, whose running performance was superior, would show less fluctuation in pacing than Group B, no such tendency was observed. We were able to demonstrate that there was no significant difference in pacing between the two groups. The results of this study may be of great value for collegiate triathletes to compete in elite races. Further evaluation at a wide range of competition levels is required in future studies.

## Data Availability Statement

The raw data supporting the conclusions of this article will be made available by the authors, without undue reservation.

## Ethics Statement

Permission to obtain video data was obtained from the competition staff. At the competition briefing, the staff explained to the participating athletes the nature of this survey and explained that they could withdraw if they were unable to participate.

## Author Contributions

YI and AA came up with the idea for the study. YI, AA, and HN organized the data. YI performed statistical processing and created the first edition of the dissertation. AA, KI, and HN collected data and created the last version of the dissertation. YN and TA assisted in the preparation of the final version of the dissertation. All authors contributed to the dissertation and approved the submitted version.

## Funding

This work was supported by JST SPRING, Grant Number JPMJSP2128.

## Conflict of Interest

The authors declare that the research was conducted in the absence of any commercial or financial relationships that could be construed as a potential conflict of interest.

## Publisher's Note

All claims expressed in this article are solely those of the authors and do not necessarily represent those of their affiliated organizations, or those of the publisher, the editors and the reviewers. Any product that may be evaluated in this article, or claim that may be made by its manufacturer, is not guaranteed or endorsed by the publisher.
